# Management and Long-Term Follow-Up of Hyperparathyroidism in Multiple Endocrine Neoplasia Type 1: Single Center Experience

**DOI:** 10.3390/jcm11071967

**Published:** 2022-04-01

**Authors:** Maria P. Yavropoulou, Sofia Vlachou, Marina Tsoli, Florentia Fostira, Gregory Kaltsas, Eva Kassi

**Affiliations:** 1 Endocrinology Unit, First Department of Propaedeutic and Internal Medicine, Centre of Expertise for Rare Endocrine Disease (C.E.R.E.D.), Medical School, National and Kapodistrian University of Athens, 11527 Athens, Greece; sophie-vl@hotmail.com (S.V.); martso.mt@gmail.com (M.T.); gkaltsas@med.uoa.gr (G.K.); ekassi@med.uoa.gr (E.K.); 2 Molecular Diagnostics Laboratory, National Center for Scientific Research “Demokritos”, Institute of Nuclear & Radiological Sciences & Technology, Energy & Safety, 15341 Athens, Greece; florentia@rrp.demokritos.gr

**Keywords:** primary hyperparathyroidism, MEN1, long-term follow-up, cinacalcet, permanent hypoparathyroidism

## Abstract

Background: Primary hyperparathyroidism (PHPT) in the most common and earliest manifestation of multiple endocrine neoplasia type-1 (MEN1). Epidemiological data have been reported in MEN1 patients but data on long-term follow-up focusing on PHPT are scarce. Methods: In this retrospective cohort study, we included patients diagnosed with MEN1-related PHPT that were under regular follow-up in our institution. Results: Data on 68 patients (39 males), with a mean age at MEN1-diagnosis of 39 ± 13.06 years, were analyzed. Pancreatic neuroendocrine tumors were encountered in 82% (71% nonsecreting) followed by pituitary adenomas in 66% (49% nonsecreting). Mean age at PHPT diagnosis was 35.2 ± 4.0 years. Parathyroidectomy was performed in 57 patients (82.3%), of whom 56% achieved long-term remission, while 12.2% and 31.5% had persistent and recurrent disease, respectively (median follow-up of 4 years; range 1–21 years). Cinacalcet restored serum calcium levels in 33.8%, both as first and as a second line treatment. Permanent hypoparathyroidism occurred in 19.2%. *MEN1* pathogenic variants were identified in 77.2% of the tested individuals, but no genotype-phenotype associations were reported. Conclusions: MEN1-related PHPT involves a multiglandular disease and its management remains a therapeutic challenge, as recurrent disease can develop even after 20 years of follow-up. Prolonged follow-up of these patients at referral centers is critical for their optimal management.

## 1. Introduction

Multiple endocrine neoplasia type-1 (MEN1) is a rare endocrine syndrome, associated with *MEN1* pathogenic variants, inherited by autosomal dominant manner, and characterized by the occurrence of primary hyperparathyroidism, pituitary adenoma, and pancreatic neuroendocrine tumors (NETs). Other endocrine and nonendocrine tumors such as adrenal tumors, duodenal, thymic and lung NETs, lipomas, facial angiofibromas, and collagenomas also develop in the context of MEN1 syndrome [1].

Inactivating variants in the tumor-suppressor gene, *MEN1*, located in 11q13 chromosome are the genetic cause of MEN1, encoded for menin. Menin is a nuclear 610 amino acid scaffold protein that is ubiquitously expressed and is involved in several cellular processes. Up to date, more than 450 different pathogenic variants have been identified, but no genotype-phenotype correlations have been identified so far [2]. The prevalence of MEN1 syndrome is estimated to be 1/30,000 to 1/50,000 [3]. However, the penetrance of MEN1 is quite high, with more than 95% of asymptomatic carriers developing at least one feature by the age of 40 years [4]. In about 10% of individuals bearing characteristics of MEN1 syndrome, no *MEN1* causative variant is identified [1], either due to the presence of structural variants, or due to the presence of variants in a gene’s promoter and untranslated regions, phenomena that might not be routinely identified. Additionally, involvement of other genes, such as the Cyclin Dependent Kinase Inhibitor 1B (*CDKN1B*) gene, might explain a number of cases [5].

Primary hyperparathyroidism (PHPT) is usually the initial clinical manifestation occurring in more than 90% of the patients. It appears at a younger age (20–25 years), is characterized by multiglandular disease, and is clinically presented with symptomatology related to hypercalcemia, although it can remain asymptomatic for a long time. Surgery is considered the treatment of choice, and the surgical approach differs from sporadic parathyroid adenomas, since involvement of more than one parathyroid gland is the characteristic feature of MEN1-related PHPT [1]. In addition, the enlargement of the glands may be asynchronous and asymmetric [6], as each gland is considered to be a monoclonal lesion [4,7]. As such, a high failure rate for parathyroidectomy is frequently reported in these patients, including higher recurrence rates as well as higher incidence of permanent hypoparathyroidism compared to PHPT due to sporadic adenomas. On the other hand, medical therapies for MEN1-related PHPT, including anti-resorptive drugs such as bisphosphonates and calcimimetics, have a limited role [8,9]. Nationwide registries with population-based research and long-term follow-up would be the best means to improve therapeutic modalities in MEN1-related PHPT, but data are scarce. In this study, we performed a retrospective analysis of clinical and genetic data of patients diagnosed with PHPT in the context of MEN1 and followed up in one single referral center in Greece.

## 2. Patients and Methods

This is a retrospective cohort study including patients diagnosed with PHPT in the context of MEN1 that were under regular follow-up in the Centre of Expertise in Rare Endocrine Diseases (C.E.R.E.D) of the Medical School, National and Kapodistrian University of Athens. Only adult patients with available clinical, biochemical, and genetic data were included in the analysis. The diagnosis of PHPT in the context of MEN1 was based on the following criteria: (1) multiglandular disease with a histopathological diagnosis of parathyroid hyperplasia (where available) and (2) occurrence of one more primary MEN1-associated endocrine tumor or occurrence of one of the MEN1-associated tumors in a first-degree relative of the patient with a clinical diagnosis of MEN1 or identification of a germline *MEN1* pathogenic variant [1]. Data of the finally enrolled patients (e.g., laboratory and pathology results and radiological imaging) were collected from the medical records of C.E.R.E.D in accordance with the General Data Protection Regulation (GDPR) policy of the Center.

As per standard clinical practice in our institution, in patients diagnosed with MEN1-related PHPT we recommend (i) parathyroid imaging (ultrasound by an expert radiologist and in some cases a ^99m^Tc-parathyroid scintigraphy) to evaluate the extent of the disease at the time of diagnosis, (ii) bone mineral density measurement by dual-energy X-ray absorptiometry (DXA) scan, (iii) renal ultrasound to investigate the presence of nephrolithiasis and/or nephrocalcinosis, and (iv) subtotal parathyroidectomy plus thymectomy by an experienced surgeon. However, since patients are frequently referred to us from other institutions, DXA scans and renal ultrasounds are not systematically performed and the choice for the extent of the parathyroid surgery may vary between patients.

Osteoporosis is diagnosed when BMD is 2.5 SD or more below the average value for young healthy women (a T-score of < −2.5 SD) and osteopenia as a T-score between −1 and −2.5 SD, based on the World Health Organization (WHO) criteria [10] for postmenopausal women and males older than 50 years. The same thresholds are also applied to younger individuals, older than 20 years and in the absence of delayed puberty, as recommended by the International Foundation of Osteoporosis (IOF) [11].

### 2.1. Statistical Analysis

The data are presented as mean ± standard deviation (SD), median, and percentage. Logistic regression was used to assess the impact of the identification of a pathogenic variant or the type of the parathyroidectomy on the occurrence of the studied clinical events. A *p* < 0.05 was considered significant. Statistical analysis was performed using IBM SPSS Statistics for Windows, Version 26 (IBM SPSS Statistics for Windows, IBM Corporation, Armonk, NY, USA).

### 2.2. Genetic Analysis

Written informed consent was provided by all individuals participating in the study. Genomic DNA was isolated from peripheral blood lymphocytes using the salt extraction protocol. The quantity and quality of DNA samples were determined by UV absorbance using a NanoDrop 1000 spectrophotometer (Thermo Fisher Scientific, Waltham, MA, USA). All *MEN1* coding exons were PCR-amplified and were further bidirectionally sequenced using the v.3.1 BigDye Terminator Cycle Sequencing kit on an ABI 3130XL Genetic Analyzer (Thermo Fisher Scientific, Waltham, MA, USA), following the manufacturer’s instructions. Primer sequences and protocols are available upon request. All samples negative for small nucleotide variants were further analyzed for large genomic rearrangements using Multiple Ligation Probe Amplification (SALSA MLPA Probemix_P244) provided by MRC Holland (Amsterdam, The Netherlands).

## 3. Results

### 3.1. Patients’ General Characteristics

**Figure 1:**
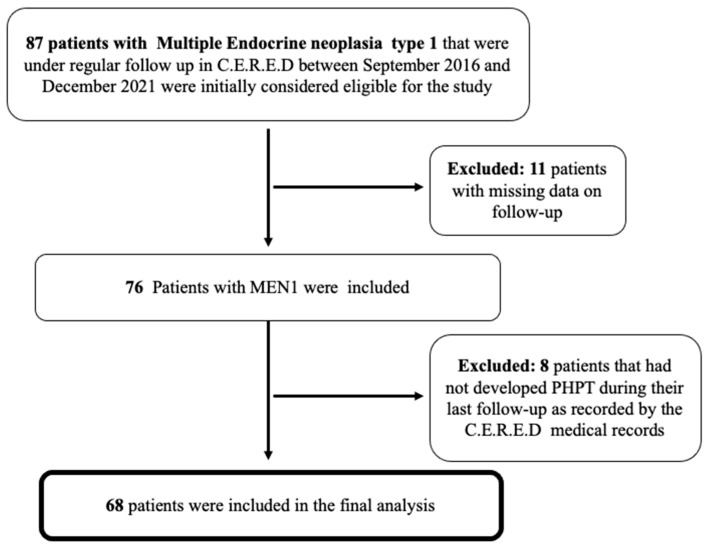
Flow chart of the finally included patients with MEN1-related PHPT that were under regular follow-up in our Centre (C.E.R.E.D). MEN1, Multiple Endocrine Neoplasia type 1; PHPT, primary hyperparathyroidism.

Our cohort consisted of 68 patients with PHPT in the context of MEN1 with a median follow-up of 4 years (range between 9 months and 21 years) (Figure 1).

Demographic characteristics and the presence of other endocrine tumors are depicted in Table 1.

Out of the 68 registered patients, 39 (57.4%) were males and 29 (42.6%) were females, with a mean age at MEN1 diagnosis of 39 ± 13.06 years. Family history of MEN1 was present in 76.5% of the patients. Primary hyperparathyroidism was the initial clinical manifestation in the majority of MEN1 patients (*n* = 44, 64.7%), while 18 patients (26.5%) developed a neuroendocrine tumor (67% of them located in the pancreas), and 6 patients (8.8%) developed a pituitary adenoma as the first MEN1-related clinical manifestation. In addition, almost 60% of the patients (*n* = 39) also developed adrenal cortical pathology, including 17 patients with uni- or bilateral adenomas sized between 1.8 to 4.4 cm, 2 patients with myelolipomas, 11 patients with uni- or bilateral hyperplasia, and 9 patients with adrenocortical thickening. The majority of adrenocortical adenomas were nonfunctioning, while three of them were classified as mild autonomous cortisol secreting (MACS) adenomas. Nonendocrine tumors were also reported in 42.6% of the patients, including 17 patients with liver lesions, of which, 10 were cystic and 7 were hemangiomas, 11 patients (16%) with angiofibromas/collagenomas, and 1 patient with a neck lipoma. Metastasis from NET tumors were evident in 12 patients (21.4%), located in the liver (*n* = 11) or the adrenal cortex (*n* = 1).

Other comorbidities were present in 72% of the patients (*n* = 49), with diabetes mellitus type 2 found more frequently (*n* = 24, 35%), followed by hypertension (*n* = 20, 29%). Fourteen patients (20.5%) also developed thyroid pathology (hypothyroidism and multinodular goiter), of whom four (5.9% of the whole patient’s cohort) were diagnosed with differentiated thyroid carcinoma. Death occurred in eight of our patients (11.7%) during long-term follow-up, with the most common cause of death being advanced liver disease due to metastasis from neuroendocrine tumors.

### 3.2. Diagnosis and Complications of MEN1-Related Primary Hyperparathyroidism

The mean age at PHPT diagnosis was 35.2 ± 14.0 years, with only 13.2% of them (*n* = 9) being diagnosed above the age of 50 years (mean age 57.2 ± 6.5). In most of the cases, the initial manifestation was biochemically diagnosed hypercalcemia or nephrolithiasis, while none of the patients had sustained a fragility fracture. Preoperatively, mean serum calcium levels adjusted for albumin and mean serum PTH levels were 11.2 ± 0.9 mg/dL (normal values between 8.4–10.4 mg/dL) and 153.4 ± 92 pg/mL (normal values between 14–65 pg/mL), respectively.

Eleven patients (17.6%), five males and six females, had developed osteoporosis during the course of the disease, with a mean age at diagnosis of 57.7 ± 9 years. In addition, four patients (two premenopausal females and two males) with a mean age 30.25 ± 4.2 years were also diagnosed with osteopenia during screening for BMD measurement at the time the diagnosis of MEN1-related PHPT was established in our institution. In 34 patients that were examined with renal ultrasound after the diagnosis of PHPT, 22 (64.7%) had nephrolithiasis and 1 had nephrocalcinosis. Data available from 36 patients that had a parathyroid ultrasound revealed that 11% had no parathyroid pathology, while the majority of radiology reports described bilateral parathyroid adenomas. ^99m^Tc-sestamibi parathyroid scintigraphy was also performed as a complementary technique to thyroid ultrasound. Two out of the four patients with no pathological findings in parathyroid ultrasound had a positive ^99m^Tc-sestamibi parathyroid scintigraphy, whereas the results of both imagings were concordant in most of the cases (84.6%).

### 3.3. Management and Follow-Up of Primary Hyperparathyroidism

Parathyroidectomy (PTX) was performed in 57 of the 68 patients (83.8%). At the initial parathyroid surgery, 22 patients (38.5%) had one (*n* = 14) or two parathyroid glands resected (*n* = 8) based on the results of the parathyroid imaging (ultrasound and/or ^99m^Tc- parathyroid scintigraphy). In this group of patients, PHPT was diagnosed before the establishment of MEN1 diagnosis and were therefore managed as sporadic parathyroid adenomas before being referred to our institution. Mean age of these patients at MEN1 diagnosis was 39.4 ± 11 years, and PHPT was the initial manifestation in the majority of them (77.2%, *n* = 17); in four patients the first diagnosis was a NET and in one patient a pituitary adenoma. In the majority of our patients’ cohort (61.5%, *n* = 35), however, MEN1 diagnosis was established or highly suspected, based on clinical data and/or family history, preoperatively and therefore a subtotal parathyroidectomy with three or more glands resected was performed.

Of the 11 patients that did not undergo parathyroid surgery at the time of the data collection, 1 patient was diagnosed with MEN1-related PHPT during the first trimester of pregnancy; 1 patient (a 25-year-old female) was newly diagnosed with PHPT; and 9 patients aged between 39 to 62 years were unwilling to proceed to parathyroid surgery and were under treatment with the calcimimetic agent cinacalcet. Two patients were also treated with bisphosphonates for osteoporosis.

**Figure 2:**
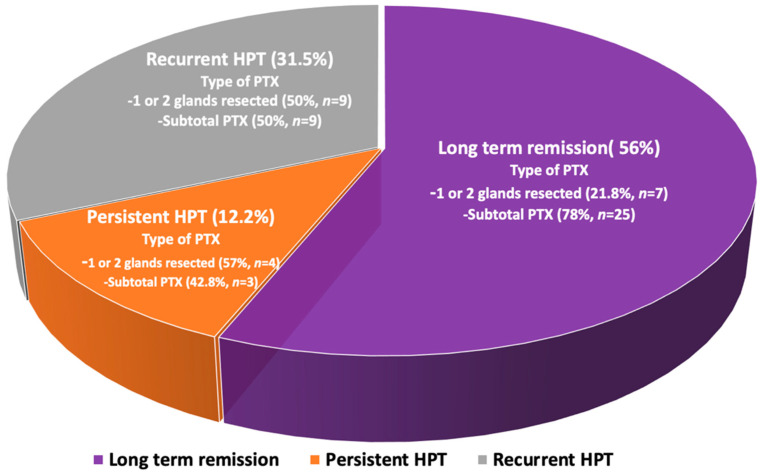
Schematic depiction of the outcome of the initial parathyroid surgery in MEN1-related PHPT.

Long-term remission of PHPT was achieved in 32 patients (56%), persistent PHPT was reported in 7 (12.2%), and recurrent disease was found in 18 patients (31.5%) at a median follow-up of 4 years (ranging between 1 and 21 years) (Figure 2), while no incidence of laryngeal nerve damage was reported. Long-term remission was strongly associated with subtotal parathyroidectomy with an OR of 1.7 (95% CI: 1.2–3.7, *p* < 0.001), compared to surgical resection of only one or two parathyroid glands.

Reoperation for recurrent disease was performed in 11 of the 18 patients (61%), of whom 4 had subtotal parathyroidectomy, 6 had one parathyroid gland resected, and 1 had two parathyroid glands resected at the initial parathyroid surgery. Permanent hypoparathyroidism occurred in 11 patients (19.2%), of whom 9 had subtotal parathyroidectomy at the initial parathyroid surgery and 2 developed hypoparathyroidism after reoperation due to recurrent disease. A total of 23 patients (33.8%) were treated with the calcimimetic agent cinacalcet with favorable results on serum calcium levels, including the 7 patients that developed recurrent disease and did not undergo a second parathyroid surgery, 7 patients with persistent hypercalcemia after the first parathyroid surgery, and 9 out of the 11 patients that did not perform parathyroid surgery (2 of them were treated with oral bisphosphonates for osteoporosis).

### 3.4. Genetic Test Results

Genetic analysis was performed in 43 patients (63.2%); 6 were first-degree relatives of the initially tested individuals (probands). A pathogenic or likely pathogenic variant known to cause MEN1 was identified in 36 (83.7%) of them, while 1 patient carried a variant of uncertain significance (VUS) (Table 2). Notably, no large gene rearrangements (LGRs) were detected by MLPA in our patient series. All variants have been classified based on the American College of Genetics and Genomics (ACMG) criteria for variant classification [12]. No significant differences were reported in the clinical characteristics of MEN1, initial manifestations, or complications of PHPT between patients that harbor a known pathogenic *MEN1* variant versus patients with negative test results, including patients with VUS.

## 4. Discussion

Primary hyperparathyroidism in the context of MEN1 involves a multiglandular disease with small hyperplastic glands rather than adenomas, which remains challenging for most imaging modalities and surgery techniques. Multi-center studies that analyzed large groups of patients as well as nation-based single-center experiences have been reported before [13,14,15,16] in MEN1 patients, but data on the long-term follow-up of these patients focusing exclusively on PHPT are scarce.

In line with current knowledge [14], most of the patients in our cohort developed PHPT before the age of 50 (86.7%), and hyperparathyroidism was the earliest endocrine manifestation of MEN1 in 64% of them. Additionally, in accordance with other registries, primary manifestations of MEN1-related PHPT were biochemically diagnosed as hypercalcemia and nephrolithiasis [13,14,15,17]. In addition, in a significant proportion of patients (approximately 70%) in whom nephrolithiasis was not evident upon diagnosis, it developed during the course of the disease, highlighting the importance of continuous monitoring of these patients.

Although imaging is not considered necessary for the diagnosis of parathyroid disease in MEN1-related PHPT for known multiglandular involvement, parathyroid ultrasound may provide valuable information about the extension of the disease at the time of the examination and the detection of ectopic as well as supernumerary glands [18]. In our cohort of MEN1 patients, parathyroid ultrasound and ^99m^Tc-sestamibi parathyroid scintigraphy were diagnostic for multiglandular diseases in most of the cases where data were available. Two out four of the patients with no abnormal findings in parathyroid ultrasound had a positive ^99m^Tc-sestamibi parathyroid scintigraphy. On the other hand, in a considerable number of MEN1 patients, the parathyroid ultrasound may be misleading, showing one affected parathyroid gland, and thus delaying the diagnosis of MEN1-related PHPT. In our center, as well as other institutions in Greece, it is common practice that patients with PHPT, independent of the underlying cause, are referred to parathyroid imaging with ultrasound by expert radiologists in the field. Nevertheless, since the technique is subjective and largely dependent on the experience of the radiologist, negative reports in cases with high clinical suspicion of MEN1 should be treated with caution.

Several studies have proposed preoperative localization procedures that are more sensitive to detect multiglandular disease, small lesions, or possible ectopic parathyroid tissue in MEN1-related PHPT, such as 18F-fluorocholine PET/CT (FCH-PET/CT), with or without enhanced arterial imaging, and four-dimensional computed tomography (4D-CT), especially when conventional preoperative imaging before the first intervention is inconclusive [19,20,21,22]. In particular, the performance of 18F-FCH-PET/CT in PHPT appears to be superior compared to commonly used imaging using ^99m^Tc-sestamibi scintigraphy, ultrasound, and 4D-CT [21,23,24,25,26], and provides valuable information even in cases with recurrent disease and negative or equivocal ^99m^Tc-sestamibi scintigraphy and/or ultrasound.

Although the progression of PHPT in the context of MEN1 is usually gradual, the optimal treatment remains parathyroidectomy, with favorable results in reducing the risk of kidney stones, fragility fractures, and cardiovascular morbidity, while it has also been demonstrated that it significantly improves bone mineral density and quality of life [27]. Which surgical approach to choose between subtotal parathyroidectomy with removal of at least three to three and a half glands, and total parathyroidectomy with removal of all parathyroid glands and with subsequent autologous parathyroid tissue graft, depends on the clinical characteristics of the patient and the experience of the surgeon. Extensive surgery is burdened by an increased risk of permanent hypoparathyroidism and recurrent laryngeal nerve injury, while less extensive surgery yields a higher risk of recurrent disease requiring reintervention, which increases the risk of more surgical complications [14,28].

In our cohort, parathyroidectomy was performed in 82% of the MEN1 patients, of whom the majority underwent subtotal parathyroidectomy, while we had no cases of total parathyroidectomy with autologous parathyroid tissue graft. However, a considerable proportion of patients, almost 40%, underwent excision of only one or two parathyroid glands based on their imaging results, especially in those in whom the diagnosis of PHPT preceded the diagnosis of MEN1 and were therefore managed as sporadic PHPT.

Despite the presence of an experienced MEN1 surgeon, which is strongly recommended in these cases [27], persistent or recurrent disease remains a significant and challenging issue. In our cohort, despite the performance of subtotal parathyroidectomy in most of our cases (61%,) the rates of persistent and recurrent disease remained high (in nearly 40% of our patients that underwent parathyroidectomy), further complicating the management of these patients.

The rate of persistent disease in MEN1-related PHPT in the hands of an expert surgeon is documented to be less than 20%, although it may rise to 40–60% for less experienced surgeons [27]. In our patients, persistent disease accounted for 12% and mostly included cases that underwent excision of only one or two parathyroid glands. The rate of recurrent disease on the other hand, although no consistent data are available, is reported to range between 2.5 and 9.8% [29] and can be developed up to 20 years after the initial parathyroid surgery, as we report in one of our patients. In these cases, localization for possible ectopic parathyroid tissue, using advanced imaging modalities such as 18F-fluorocholine PET/CT, should be considered [30], and long-term, yearly monitoring of calcium concentrations after a successful parathyroid surgery is strongly recommended [30].

The management of persistent or recurrent disease mainly depends on the patient’s clinical status and the experience of the center. The European Society of Endocrine Surgeons suggests that challenging procedures, such as reinterventions for PHPT, should only be performed in highly experienced centers regarding reoperative parathyroid surgeries [31] with available, if possible, surgical adjuncts, such as intraoperative PTH assay and intraoperative nerve-monitoring [30]. In our cohort, 11 out of the 18 patients (61%) that developed recurrent disease underwent reoperation with remission of hypercalcemia and no reported PTX-related complications except for one case that developed permanent hypoparathyroidism. The rest of the patients *(n* = 7) were managed conservatively with calcimimetics. Both therapeutic approaches sufficiently restored serum calcium levels, but data on long-term follow-up are missing.

Conservative medical management using calcimimetics and bone protecting agents remains an alternative therapeutic option in MEN1-related PHPT especially in patients with mild disease and/or severe comorbidities [28], albeit with limited data available on the long-term. Cinacalcet is the only currently available calcimimetic agent that lowers serum calcium and PTH by increasing the sensitivity of the calcium sensing receptor (CaSR) to extracellular calcium, which in turn decreases serum PTH and renal tubular reabsorption of calcium. It is reported to normalize serum calcium in 70–80% of patients with PHPT due to sporadic adenomas [32] in cases where surgery is not an option, but data on MEN1-related PHPT are scarce. In our cohort, cinacalcet was used both as a first-line treatment in patients that did not have parathyroid surgery and as a second-line treatment in patients with persistent or recurrent parathyroid disease. All patients had favorable results in calcium-level restoration even in the long-term (followed up to 13 years) without serious adverse events reported, but long-term data on BMD measurements and renal complications are missing. Two of our patients that were treated with only oral bisphosphonates had also maintained serum calcium levels in the upper normal range, in line with previous reports and guidelines [28,33,34].

Pathogenic *MEN1* variants are distributed throughout the coding region of the gene and to date, there are no consistent genotype-phenotype correlations, as opposed to mutations in the *RET* proto-oncogene [35]. In our cohort, causative variants for MEN1 were identified in the vast majority of the patients tested, i.e., 83,7%, while one patient carried a VUS. We have not been able to identify a consistent pattern to imply a genotype-phenotype association, although our numbers were small.

## 5. Conclusions

PHPT in the context of MEN1 involves a multiglandular disease and remains a therapeutic challenge over the long-term for most treating physicians, as recurrent disease after parathyroid surgery can develop even after 20 years of follow-up. Calcimimetic agents are an efficient alternative approach both as a first-line and as a second-line treatment in unoperated patients and in patients with persistent or recurrent disease after parathyroidectomy, respectively, but long-term data on BMD measurements and the incidence of renal complications are missing. The need for nationwide cohort studies and national registries of rare diseases has become very well established in the last decade. Collection of clinical, biochemical, and genetic characteristics of MEN1 in referral centers at a national level with a multidisciplinary approach is critical for the optimal care of these patients.

## Figures and Tables

**Table 1:** Clinical characteristics of the patients’ cohort (*n* = 68).

Males, *n* %	39 (57.4%)	
Family history, *n* %	52 (76.5%)	
Pituitary adenomas, *n* %	45 (66.2%)	Classification of pituitary adenomas, *n* % Nonsecreting: 22 (48.8%) Prolactinomas: 20 (44.4%) Corticotropinomas: 1 (2.2%) Somatotropinomas: 2 (4.4%)
	
Microadenomas	33 (73%)	
Macroadenomas	12 (26%)	
Neuroendocrine tumors, *n* %	56 (82.3%)	Classification of Neuroendocrine tumors, *n* % Nonsecreting: 40 (71.4%) Pancreatic: 38 (67%) Gastrinomas: 13 (23.2%) Insulinomas: 4 (7.1%) One patient developed both an insulinoma and a gastrinoma
	
Single lesion	23 (41%)	
≥2 lesions	33 (58.9%)	
Metastatic lesions	12 (21.4%)	
Adrenal Tumors	39 (57.3%)	
Liver lesions	17 (25%)	
Subcutaneous lesions	12 (17.6%)	

Data are presented as number of patients *n* (%).

**Table 2:** Summary of identified variants *MEN1*.

Gender, (Age at MEN1 Diagnosis)	HGVS Nomenclature	Significance
Male (39 years old)	c.1A>G	Pathogenic variant
Male (53 years old)	c.2T>A	Likely pathogenic variant
Male (24 years old) *	c.2T>A	Likely pathogenic variant
Male (37 years old)	c.247_250del	Pathogenic variant
Male (21 years old)	c.493T>C	Pathogenic variant
Male (39 years old)	c.494G>A	Pathogenic variant
Male (47 years old) *	c.494G>A	Pathogenic variant
Male (44 years old)	c.628_631del	Pathogenic variant
Male (36 years old)	c.685G>A	Pathogenic variant
Female (63 years old)	c.778G>A	Pathogenic variant
Female (30 years old)	c.778G>A	Pathogenic variant
Female (23 years old)	c.799-9G>A	Pathogenic variant
Male (14 years old) *	c.799-9G>A	Pathogenic variant
Male (39 years old)	c.861A>C	Pathogenic variant
Female (34 years old)	c.958del	Pathogenic variant
Male (41 years old)	c.1096G>T	Pathogenic variant
Male (29 years old)	c.1110_1111delins	Pathogenic variant
Male (24 years old)	c.1235dup	Pathogenic variant
Male (62 years old)	c.1259_1260delins	Pathogenic variant
Female (16 years old)	c.1270dup	Pathogenic variant
Male (37 years old) *	c.1270dup	Pathogenic variant
Female (36 years old)	c.1270dup	Pathogenic variant
Female (50 years old) *	c.1270dup	Pathogenic variant
Male (27 years old)	c.1270dup	Pathogenic variant
Female (46 years old)	c.1270dup	Pathogenic variant
Female (53 years old)	c.1285dup	Pathogenic variant
Male (26 years old)	c.1285dup	Pathogenic variant
Male (29 years old) *	c.1285dup	Pathogenic variant
Female (47 years old)	c.1351-3_1359del	Pathogenic variant
Male (34 years old)	c.1371_1382del	Pathogenic variant
Female (39 years old)	c.1378C>T	Pathogenic variant
Male (50 years old)	c.1421delinsCG	Pathogenic variant
Female (52 years old)	c.1546_1547insC	Pathogenic variant
Female (32 years old)	c.1546_1547insC	Pathogenic variant
Male (44 years old)	c.1561dup	Pathogenic variant
Male (38 years old)	c.1675C>T	Pathogenic variant
Male (46 years old)	c.434_439del	Variant of uncertain significance

* First degree relatives of probands. All variants described are based on Reference Sequence NM_000244.3.

## Data Availability

Not applicable.
